# Descriptions of advanced multimorbidity: A scoping review with content analysis

**DOI:** 10.1177/26335565251326309

**Published:** 2025-03-18

**Authors:** Sarah P Bowers, Polly Black, Lewis McCheyne, Darcy Wilson, Rose S Penfold, Liam Stapleton, Pam Channer, Sarah E E Mills, Linda Williams, Frances Quirk, Jo Bowden

**Affiliations:** 1 School of Medicine, 7486 University of St Andrews, St Andrews, UK; 2 3049 NHS Fife, Fife, UK; 3 1251 NHS Tayside, Dundee, UK; 4 Ageing and Health, Usher Institute, 59892 University of Edinburgh, Edinburgh, UK; 5 Advanced Care Research Centre, 59892 University of Edinburgh, Edinburgh, UK; 6 8964 University College London Hospitals NHS Foundation Trust, London, UK; 7 School of Medicine, Fife Community Advisory Council, 7486 University of St Andrews, St Andrews, UK; 8 Edinburgh Clinical Trials Unit, Usher Institute, 59892 The University of Edinburgh, Edinburgh, UK

**Keywords:** Multimorbidity, scoping review, end of life, palliative care, ageing

## Abstract

**Introduction:**

Multimorbidity is associated with adverse clinical outcomes, including increased symptom burden and healthcare utilisation, particularly towards the end of life. Despite this, there is no accepted method to identify the point at which individuals with deteriorating health due to long-term conditions are nearing the end of life or might benefit from a palliative care approach – conceptualised as ‘Advanced Multimorbidity’. This scoping review explored how Advanced Multimorbidity is described and operationalised within the literature.

**Methods:**

Multiple electronic databases and Grey Literature sources were searched following scoping review frameworks. Two reviewers independently performed screening and data extraction. Content analysis was used to examine the different descriptions of Advanced Multimorbidity. Stakeholder consultations were undertaken with clinicians, academics and public participants. Patient and public involvement was separately integrated throughout this review from conceptualisation, design and reporting.

**Results:**

Forty-four different descriptions of Advanced Multimorbidity were identified from 38 publications. These varied in terms of the clinical conditions and descriptors used. Eighteen descriptions relied on a single indicator to identify Advanced Multimorbidity; 24 used a multidimensional approach. Stakeholder consultations highlighted the need for descriptions that are user-friendly and actionable.

**Conclusion:**

The lack of a standardised definition of Advanced Multimorbidity risks variance in clinical and research practice, potentially affecting patient care. A consensus on defining Advanced Multimorbidity would enable better identification of patients who could benefit from a palliative care approach, ensuring more consistent and person-centred care, as well as supporting research and policy development.

## Introduction

With the rising prevalence of people living with two or more long-term conditions, more commonly referred to as multimorbidity, there are concerns that healthcare services organised by specialism are not equipped to meet the needs of this growing population.^1,2^ Multimorbidity is associated with a range of adverse health outcomes, lower quality of life and increased utilisation of healthcare services throughout the life course.^3–6^ With increasing numbers of people *living* with multimorbidity, it follows that many more people are *dying* with and from multimorbidity.^7^ This population experiences a wide range of symptoms, significantly impacting their quality of life as their health deteriorates.^8,9^ Healthcare utilisation towards the end of life for people with multimorbidity has been shown to be fragmented, punctuated with frequent unscheduled care contacts, and costly.^10–12^ The seemingly unpredictable nature of the multimorbidity illness trajectory makes identifying the end-of-life period challenging. A lack of consistent and proactive identification risks denying this group of patients and their caregivers honest conversations about their mortality and the opportunity to participate in future care planning with a view to aligning their care with what matters most to them – a key component of a palliative care approach which can be delivered by generalists and specialists alike.^13,14^

Increasingly it is being demonstrated palliative care is no longer exclusively a model of care for those at the very end of life, with a growing evidence base of the benefits of earlier integration of palliative approaches.^15^ However, the optimal timing of when to integrate palliative care into the care of those with multiple health conditions remains unclear. Such identification and action could not only improve the appropriateness of care received by people whose life expectancy is likely to be limited, through the avoidance of potentially harmful, ineffective or unwanted treatments; but may also, from a health service perspective, increase the capacity to provide curative and preventative interventions for those more likely to benefit. In order to make a difference to the quality and nature of care that people with multimorbidity experience towards the end of life, we must be able to reliably identify the time when a palliative care approach should be considered and offered.^16^

At present, there is no accepted definition to support clinical identification of when people with multimorbidity would benefit from a palliative care approach or are approaching the end of their lives. Advanced Multimorbidity is a term that could reasonably describe an individual with progressively deteriorating health due to their multiple long-term conditions.^17^ Understanding how best to identify people with Advanced Multimorbidity would allow for a more reliable and coordinated approach to care which optimises quality of life with simultaneous planning for future deterioration.

## Scoping review aim

This scoping review aimed to collate the available evidence on how people with Advanced Multimorbidity are described within the literature, with a view to identifying gaps in knowledge and making recommendations for future research.

## Methods

This scoping review adhered to the Arksey and O’Malley framework for scoping reviews while referencing the Joanna Briggs Institute guidance and updates from Levac et al. – including refinement of the research question, ensuring appropriate expertise within the review team, and incorporating a more robust stakeholder consultation.^18–20^ It is reported in accordance with the Preferred Reporting Items for Systematic Review and Meta-Analyses extension for Scoping Reviews (PRISMA-ScR).^21^ The protocol for this review has been published.^22^

Scoping review methodology was chosen for this topic, given its role in conceptual mapping in healthcare, particularly in areas without universally agreed definitions.^23^ In accordance with the scoping review guidance,^18–20^ this review followed the five stages detailed below.

### Stage 1 – Identifying the research question

Advanced Multimorbidity was operationalised as the time period when the care of people with multimorbidity might usefully begin to transition from aiming to reverse or treat chronic conditions alone to encompass alleviating suffering and addressing multidimensional needs – in line with a ‘palliative care approach’.^16^ This description of Advanced Multimorbidity is in accordance with research into other advanced illnesses^24–26^ and aligns with previously described definitions of Advanced Multimorbidity.^17,27^

Inclusion and exclusion criteria were developed using the Population-Concept-Context model^20^ – Table 1. Studies were excluded if the primary focus was a primary index condition and/or comorbidity, as the former suggests a more specified single disease-focussed approach; whilst multimorbidity suggests a person-centred approach for individuals with multiple long-term health conditions.^28^ For research papers, only peer-reviewed, published articles were included to improve the quality of the review, as quality assessment is not a routine part of scoping review methodology.^19^ All study types were considered, including editorials and case studies, as it is recognised that these often capture details of current clinical practice that may not be represented in research.

**Table 1.** Inclusion & exclusion criteria.

Inclusion	Exclusion
Population	
Adults (>18) with multimorbidity (≥2 health conditions) or their carers or healthcare providers	Studies focusing on one index condition Studies focusing on comorbidity
Concept	
Descriptions of multimorbidity in a terminal phase of the illness experience	Studies related solely to prognosis No clear description for advanced multimorbidity in the study/report
Context	
All settings and countries Original research articles and review articles	Unpublished research Not English language

### Stage 2 – Identifying relevant studies

A search strategy was developed collaboratively with the University of St Andrews library service – Supplemental File 1. This comprised search terms relating to multimorbidity, advanced illness and palliative care. The databases Medline, EMBASE, CINAHL, Scopus and PsychInfo were searched for published articles. A Grey Literature search took place to identify unpublished work, indexed theses, clinical guidelines and policies. Databases were searched from inception through to date of first search (22^nd^ May 2023). Searches were updated on 11^th^ March 2024. Searches were limited to the English language. Forward and backward citation searching was performed in all final included reports.

### Stage 3 – Study selection

Searches were combined and deduplicated using EndNote software. Titles and abstracts were independently screened by two authors (SPB and either LM or DW). All full texts were independently reviewed by SPB with secondary review by PB, LM or DW. Any discrepancies were reviewed by a third reviewer and discussions held to reach consensus.

### Stage 4 – Charting the data

Data were extracted from each included report by two independent reviewers (SPB and PB, LM or DW) into an Excel spreadsheet, inductively. This comprised details of the citation, design and population as well as the description of Advanced Multimorbidity derived from the report.

### Stage 5 – Collating, summarising and reporting the results

Study characteristics were descriptively reported. Content analysis allowed for analysis of descriptions across different source material types (research studies and Grey Literature) to make reproducible and valid inferences.^29,30^ An inductive content analysis approach was chosen as this allows for categorisation when there is limited prior knowledge about the phenomenon of choice, in this case, a lack of definition of Advanced Multimorbidity.^30^ The content analysis process is described in Supplemental File 2.

### Stakeholder consultation

Stakeholder consultations are now seen as a crucial component to scoping reviews.^19^ Consultation exercises were held with clinicians, academics and public advisors to interrogate the initial findings of the review and to develop some consensus around concepts of Advanced Multimorbidity and the utility of these. These exercises included:

• Clinicians and academics consulted during a 2024 Academic Departments of General Practice in Scotland (ADEGS) conference presentation using electronic surveys to prompt discussions during and after the presentation.
• An online focus group with our Public Advisory Group, comprising of six public advisors with a variety of lived experiences as bereaved caregivers, to ensure that the research was meaningful to their lived experiences.

Both stakeholder events involved the lead author (SPB) presenting the scoping review findings and asking delegates to provide feedback, followed by open discussion. Findings were brought to wider scoping review team meetings which included Public Advisory Group members, to drive the focus of the analysis and interpretation.

### Patient & public involvement

In addition to the stakeholder consultation exercises undertaken, the Public Advisory Group for this project contributed to designing the review question, commented on and helped to shape the reporting of the review results and contributed to the manuscript – with one member (PC) listed as a contributing author on behalf of the group.

## Results

### Study selection

**Figure 1.**
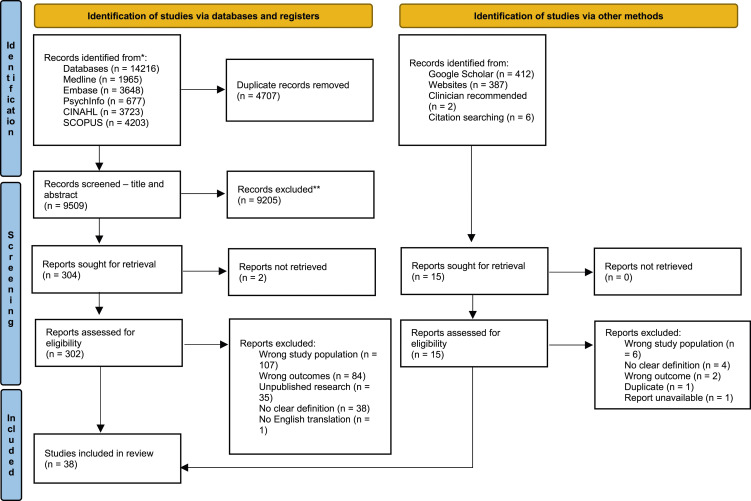
PRISMA flowchart for Scoping Reviews.

A total of 10,316 unique publications were identified – 9,509 by literature search, 801 by Grey Literature search and 6 by citation searching. After title and abstract screening, 323 publications remained. Full-text review led to a total of 38 final included publications (Table 2), which contained a description of Advanced Multimorbidity. This process is detailed in the PRISMA flow chart (Figure 1).

**Table 2.** Study characteristics.

First author surname and year	Title	Continent of study	Article methodology	Aim of article
Amblas-Novellas, 2021	Frailty degree and illness trajectories in older people towards the end-of-life: A prospective observational study	Europe	Quantitative	“In this study, aimed at improving the care of end-of-life older people, we assessed the degree of frailty in a geriatric cohort with different advanced diseases and its relationship with end-of-life illness trajectories and survival.”
Bekker, 2022	Advance care planning in primary care: a retrospective medical record study among patients with different illness trajectories	Europe	Quantitative	“Our aim was to examine the documentation of ACP for patients with cancer, organ failure and multimorbidity in medical records (as a proxy for ACP application) in Dutch general practice.”
Bernabeu-Wittel, 2023	Competences of internal medicine specialists for the management of patients with multimorbidity. EFIM multimorbidity working group position paper	Europe	Editorial	“This position paper proposes a set of emerging and re-emerging competences for internal medicine specialists, which are needed to optimally address multimorbidity now and in the future.”
Bone, 2016	Developing a model of short-term integrated palliative and supportive care for frail older people in community settings: Perspectives of older people, carers and other key stakeholders	Europe	Quantitative	“We aimed to build consensus on three key components of the model (of short term integrated palliative care): Potential benefit; timing of delivery and integrated professional working practices. Objectives were to elicit and synthesise perspectives from older people, carers and other key stakeholder to inform model development prior to a feasibility evaluation in clinical practice”
Calam, 2011	The role of specialist palliative care in managing patients with multimorbidity	Europe	Case report	“This case report describes a patient with multiple morbidity resulting from complicated type 2 diabetes, psoriatic arthritis and abdominal surgery. It highlights the importance of specialist palliative care services in meeting his complex holistic care needs.”
Clarke, 2012	‘The calendar is just about up’: Older adults with multiple chronic conditions reflect on death and dying	North America	Qualitative	“This article examines the ways in which older adults with multiple chronic conditions talk about and prepare for death and dying”
Cohn, 2018	Advanced serious illness, multimorbidity, and multibeneficence: The role of communication	North America	Editorial	“In this commentary, I will be focusing on 3 perspectives: 1) a preterminal phase of multimorbidity that is indicative of that loss of reparative or even homeokinetic properties, known by some as “advanced serious illness” 2; 2) the manifestations of advanced serious illness multimorbidity that, using the same networks that connect into the patient, are signs of this syndrome at the levels of the immediate family/friend social network, the broader community, and society at large; and 3) the potential for these same networks that transmit pathological forces to convey the positive effects of therapeutic interventions in a scale‐free manner, with a focus on how conversation can lead to what I’m calling “multibeneficence.”
Dahlqvist, 2019	Does comprehensive geriatric assessment (CGA) in an outpatient care setting affect the causes of death and the quality of palliative care? A subanalysis of the age-FIT study	Europe	Quantitative	“Does comprehensive geriatric assessment (CGA) affect the causes of death and the quality of palliative care when patients receive care at the end of life when in an outpatient care setting compared to usual care?”
De Decker, 2014	Do not resuscitate orders and aging: Impact of multimorbidity on the decision-making process	NA	Review – systematic	“To systematically review and quantitatively synthesize the evidence connecting DNR orders with multimorbidity in older patients.”
Etkind, 2022	Total uncertainty: a Systematic review and thematic synthesis of experiences of uncertainty in older people with advanced multimorbidity, their informal carers and health professionals	Australasia, Europe, North America	Review - systematic	“We aimed to identify and synthesise systematically the experience of uncertainty in advanced multimorbidity from patient, carer and professional perspectives.”
Evans C, 2021	Community-based short-term integrated palliative care for older people with chronic noncancer conditions: A randomised controlled mixed method trial	Europe	Mixed methods	“To evaluate the impact of the short-term integrated palliative and supportive care intervention for older people living with chronic noncancer conditions and frailty on clinical and economic outcomes and perceptions of care”
Evans J, 2021	Building capacity for palliative care delivery in primary care settings: Mixed-methods evaluation of the INTEGRATE project	North America	Mixed methods	“To evaluate an intervention aimed at building capacity to deliver palliative care in primary care settings.”
Finucane, 2021	How many people will need palliative care in Scotland by 2040? A mixed-method study of projected palliative care need and recommendations for service delivery	Europe	Mixed methods	“The aim of this study was to estimate, and project future palliative care need and complexity of need in Scotland up to 2040.”
Gabbard, 2021	Effectiveness of a Nurse-led Multidisciplinary intervention vs usual care on advance care planning for Vulnerable older adults in an accountable care Organization	Europe	Quantitative	“To determine whether a nurse navigator–led ACP pathway combined with a health care professional–facing EHR interface improves the occurrence of ACP discussions and their documentation within the EHR”
Gómez-Batiste, 2014	Prevalence and characteristics of patients with advanced chronic conditions in need of palliative care in the general population: a cross-sectional study	Europe	Quantitative	“Determine, by direct measurement, the prevalence of people in need of palliative care among advanced chronically ill patients in a whole geographic population.”
González González, 2020	End-of-life care preferences of older patients with multimorbidity: Protocol of a mixed-methods systematic review	Asia, Europe, North America	Review - systematic	“we systematically reviewed EoL care preferences in older patients with multimorbidity.”
Hung, 2013	Potential benefits of palliative care for polysymptomatic patients with late-stage nonmalignant disease in Taiwan	Asia	Quantitative	“Our aim was to document the polysymptomatic presentation of illness in Taiwanese patients with late-stage nonmalignant disease and to evaluate the potential benefits of palliative care for these patients”
Izumi, 2018	Feasibility and acceptability of Nurse-led primary palliative care for older adults with chronic conditions: A Pilot study	North America	Quantitative	“To assess feasibility and acceptability of a nurse-led Transitional palliative care (TPC) program for older adults with serious illness
Kelley, 2014	Identifying the population with serious illness: The “denominator” challenge	North America	Case study	“...an acronym was developed to help nurses identify appropriate residents to refer for a palliative care consult. In this article, we will discuss the acronym and a case report of an elderly male with multiple chronic incurable conditions who did not have significant weight loss”
Kelley, 2018	Does this patient in long-term care (LTC) need a palliative care consult?	North America	Mixed methods	“To define and operationalize a description of serious illness for the purpose of identifying patients and caregivers who need primary or specialty palliative care services.”
Kinzbrunner, 1996	Hospice medicine. Debility, unspecified: a Terminal diagnosis	North America	Quantitative	“To determine whether the diagnosis “debility, unspecified” (ICD-9 code 799.3) is appropriate for use with terminally ill patients and to define the criteria for assigning the diagnosis.”
Lakin, 2020	Earlier identification of seriously ill patients: an implementation case series	North America, Europe	Quantitative	“we aim to explore how diverse systems apply patient selection in practice and present a narrative case series of patient selection strategies and experiences of health systems implementing the serious illness care Program (targeted palliative care intervention)”
Llop-Medina, 2022	Palliative care in older people with Multimorbidities: A scoping review on the palliative care needs of patients, carers, and health professionals	Africa, asia, australasia, Europe, North America	Review – scoping	“The aim of this scoping review was to identify the PC needs of patients with multimorbidities (excluding cancer), their family caregivers and the professionals that work with these patients to obtain a global vision of the actors involved in the provision of PC.”
Llop-Medina, 2022	The experiences and views on palliative care of older people with Multimorbidities, their family caregivers and professionals in a Spanish Hospital	Europe	Qualitative	“Sought to understand what constitutes successful specialist palliative care in the view of all stakeholders involved, older multimorbid patients with non-cancer diseases, their informal caregivers and the health professionals who carry out their work in the field of palliative care”
Mason, 2016	‘My body’s falling apart.’ understanding the experiences of patients with advanced multimorbidity to improve care: serial interviews with patients and carers	Europe	Qualitative	“We report the experiences and perceptions of people with advanced multimorbidity to inform improvements in palliative and end-of-life care”
Mason, 2018	Computer screening for palliative care needs in primary care: a mixed-methods study	Europe	Mixed methods	“To refine and evaluate the utility of a computer application (AnticiPal) to help primary care teams screen their registered patients for people who could benefit from palliative care.”
Mayr, 2021	Feasibility of a Home-Based palliative care intervention for elderly Multimorbid Survivors of critical illness	North America	Quantitative	“To evaluate the operational feasibility of a 90-day home-based palliative care intervention in multimorbid elderly Veteran survivors of critical illness.”
Murali, 2021	Latent Class analysis of symptom burden among seriously Ill adults at the end of life	Australasia, North America	Quantitative	“The objectives of this study were to (a) describe the demographic and baseline characteristics of seriously ill adults at the end of life in this palliative care cohort; (b) identify latent subgroups of seriously ill individuals based on severity of symptom burden; and (c) examine variables associated with latent subgroup membership, such as QOL, functional status, and the presence of multiple chronic conditions.”
Nicholson, 2018	What are the main palliative care symptoms and concerns of older people with multimorbidity?-a comparative cross-sectional study using routinely collected Phase of illness, Australia-modified Karnofsky Performance status and integrated palliative care outcome Scale data	Europe	Quantitative	“To describe the clinical characteristics, symptoms and other concerns of older people with multi-morbidity referred to a new community palliative care service; and to explore possible implications for service delivery by comparing this service population with people receiving standard community-based specialist palliative care.”
Nicholson, 2023	Addressing inequity in palliative care provision for older people living with multimorbidity. Perspectives of community-dwelling older people on their palliative care needs: A scoping review	Asia, australasia, Europe, North America, South America	Review - scoping	“To understand the palliative care needs of community-dwelling people aged ⩾60 living with multimorbidity in the last 2 years of life.”
Portz, 2017	High symptom burden and low functional status in the setting of multimorbidity	Australasia, North America	Quantitative	“This analysis investigated the relationship between multimorbidity, symptom burden, and functional status in individuals with life-limiting illness.”
Powell, 2021	Palliative care for older adults with multimorbidity in the time of COVID 19	North America	Editorial	“In this article, we consider how palliative care specialists can help support this population and review how palliative care can be accessed by outpatients and inpatients during this pandemic.”
Reinke, 2019	Symptom burden and palliative care needs among high-Risk Veterans with multimorbidity	North America	Quantitative	“We assessed symptom burden and quality of life among veterans with multimorbidity and sought to determine if their bothersome symptoms were addressed and treated in the primary care setting.”
Schonfeld, 2012	Assessing challenges in end-of-life conversations with elderly patients with multiple morbidities	North America	Qualitative	“we conducted focus group interviews with primary care physicians and house staff to ask them about their experiences with elderly patients who had multiple comorbidities (MCM)”
Stokes, 2017	Does the impact of case management vary in different subgroups of multimorbidity? Secondary analysis of a quasi-experiment	Europe	Quantitative	“To explore whether the effects of case management vary in patients with different types of multimorbidity, we extend an existing analysis using these different descriptions of multimorbidity to stratify patients within the sample.”
The University of Edinburgh, 2022	Supportive and palliative care indicators tool (SPICT™)	Europe	Guideline	“SPICT™ is used to help identify people with deteriorating health due to a new serious illness, one or multiple health conditions, or frailty in older age so they benefit from holistic assessment, future care planning (advance/anticipatory care planning) and a palliative approach to care.”
The Gold standards framework, 2022	The Gold standards framework proactive identification guidance	Europe	Guideline	“aims to enable the earlier identification of people who may need additional supportive care as they near the end of their life (see GMC and NICE description of end of life care), to include final year of life as well as final days. This includes people with any condition, in any setting, given by any care provider (not just those needing specialist palliative care), following any trajectory of decline for expected deaths”
Wijers, 2019	The disease burden Morbidity assessment in older adults and its association with mortality and other health outcomes	Europe	Quantitative	“To assess how disease burden caused by chronic conditions is related to mortality (predictive validity) and other health outcomes (convergent validity).”

### Study characteristics

Included articles were published between 1992 and 2023, with the majority (33/38) published after 2014. Eighteen studies were quantitative; five were reviews; four used mixed methods research; four were qualitative; three were editorials; and two were case studies. Additionally, two palliative care needs assessment tools were identified in the Grey Literature. No policy documents or guidelines with a description of Advanced Multimorbidity were identified.

Most studies which listed a country of recruitment of participants (or in the case of editorials, for authors) (n = 37) focussed on only one continent (n = 30) – 18 were in Europe, 11 North America and one Asia. Seven studies included multiple continents. Twenty-one studies included the age of participants with a mean age across these studies of 78.5 years. Only four studies reported patient and/or public involvement in their research.^7,27,31,32^

### Descriptions of advanced multimorbidity

There was substantial variation in the number and types of conditions included in descriptions of Advanced Multimorbidity, with 44 different descriptions identified across 38 included publications (Table 3). Only two of the descriptions were used in multiple studies.

**Table 3.** Descriptions of advanced multimorbidity.

Author & year	Definition of multimorbidity Number of conditions	Definition of advanced	Content analysis
	Types of conditions		
Amblas-Novellas, 2021	≥2	Advanced frailty, recent admission, increased care needs at home, the surprise question, patient/caregiver requesting palliative/comfort treatments only, healthcare practitioner considers patient needs palliative care, albumin <2.5g/dl, >10% weight loss, nutritional decline, functional dependence measured or perceived by healthcare practitioners, loss of 2 or more activities of daily living, persistent pressure ulcers, recurrent infections, delirium, persistent dysphagia, falls, emotional distress, 2 or more unplanned hospitalisations, needing intense continuing care at home	Healthcare, functional, nutritional, self-identified, symptoms, syndromes (falls, pressure ulcers, delirium, infections), practitioner-identified
	Unspecified		
Bekker, 2022	≥2	Retrospective identification of those ≥65 years from death records	Age
	Not cancer or organ-failure		
Bernabeu-Wittel, 2023	≥2	Refractory symptoms, severe dependence and those who died	Symptoms, functional
	Unspecified		
Bone, 2016	“Conditions”	Frail and ≥75 years	Age, functional
	Non-malignant		
Calam, 2011	“Multimorbidity”	Advanced long-term condition and/or life-limiting condition, the surprise question, poor performance status, >10% weight loss, 2 or more unplanned hospitalisations, in a care home or needing more care at home	Practitioner-identified, functional, nutritional, healthcare
	Unspecified		
Clarke, 2012	≥3	Older adults who spontaneously brought up death and dying	Age, self-identified
	Most prevalent in population		
Cohn, 2018	“Several”	Declining appetite and weight loss, declining functional status, unplanned emergency department attendance and hospitalisations, declining effectiveness of medications	Functional, nutritional, healthcare, treatment response
	Unspecified		
Dahlqvist, 2019	≥3	75 years or older and hospitalised at least 3 times over 12 months	Age, healthcare
	Unspecified		
de Decker, 2014	“Multimorbidity”	80 years or older	Age
	Unspecified		
Etkind, 2022 (1)	≥2	Presence of life-limiting conditions	Nature of conditions
	Life-limiting conditions		
Etkind 2022 (2)	≥2	Life-limiting condition and functional impairment	Nature of conditions, functional
	One must be a life-limiting conditions		
Etkind 2022 (3)	≥2	Life-limiting condition and high healthcare use	Nature of conditions, healthcare
	One must be a life-limiting conditions		
Etkind 2022 (4)	≥2	Functional impairment and high healthcare use	Healthcare, functional
	Unspecified		
Etkind 2022 (5)	≥2	Identified as approaching end of life through the surprise question or Gold standards framework criteria	Practitioner-identified
	Unspecified		
Evans C, 2021	“Conditions”	≥75 years, frail and have >2 of unresolved symptoms, increased caregiver suffering and rising healthcare use	Age, functional, symptoms, caregiver burden, healthcare
	Non-malignant		
Evans J, 2021	“Multiple”	>75 years and the surprise question	Age, practitioner-identified
	Unspecified		
Finucane, 2021	≥2	Chronic diseases associated with palliative care need	Nature of conditions
	Conditions with palliative care need		
Gabbard, 2021	Charlson ≥3	65 years or older and indication of cognitive or physical impairment and/or frailty	Age, functional
	Charlson conditions		
Gómez-Batiste, 2014	≥2	Healthcare practitioners identified advanced chronically ill patients	Practitioner-identified
	Most prevalent in population		
González González, 2020	≥2	≥60 years	Age
	Unspecified		
Hung, 2013	≥2	Particular conditions, hospital admission due to exacerbation of chronic illness, infection, gastrointestinal bleeding or shock and physicians identified a “do not resuscitate” form	Nature of conditions, practitioner-identified, healthcare, syndromes (infection, gastrointestinal bleeding or shock)
	Five different non-malignant conditions		
Izumi, 2018	≥2	≥55 and hospital admission	Age, healthcare
	Prespecified (not justified)		
Kelley, 2014	“Multiple”	Non-healing wounds, recurrent infections, progressive functional decline, >10% weight loss, frequent use of emergency department or hospitalisations or unrelieved symptoms	Syndrome (wounds, infections), functional, nutritional, healthcare, symptoms
	Unspecified		
Kelley, 2018	≥3	Dartmouth atlas list conditions, healthcare utilisation, functional dependence, nutritional decline, cognitive impairment, symptoms and caregiver strain	Nature of conditions, healthcare, functional, nutritional, symptoms, caregiver strain
	Dartmouth atlas list		
Kinzbrunner, 1996^a^	Decline not due to “one specific disease”	Terminal prognosis cannot be attributed to one specified disease	Nature of conditions
	Unspecified		
Lakin, 2020	“Multiple”	Multiple hospitalisations, particular insurance types and the surprise question	Healthcare, practitioner-identified, benefit eligibility
	Unspecified		
Llop-Medina, 2022	≥2	≥60 years	Age
	Unspecified		
Llop-Medina, 2022	“Multimorbid”	≥65 years	Age
	Unspecified		
Mason, 2016 (1)	“Multiple”	The surprise question, life-limiting conditions	Practitioner-identified, nature of conditions
	Life-limiting conditions		
Mason, 2016 (2)	“Several”	The surprise question	Practitioner-identified
	Unspecified		
Mason, 2018 (1)	“Multiple”	“Multiple organ failure” code	Nature of conditions
	Palliative care need		
Mason, 2018 (2)	“Multimorbidity”	Housebound	Functional
	Unspecified		
Mason, 2018 (3)	3	Heart failure, chronic kidney disease and chronic obstructive pulmonary disease	Nature of conditions
	Palliative care need		
Mason, 2018 (4)	2	Peripheral vascular disease and chronic kidney disease	Nature of conditions
	Palliative care need		
Mayr, 2021	“Multimorbid”	Elderly intensive care unit survivors	Age, syndrome (ICU discharge)
	Unspecified		
Murali, 2021^b^	≥2	Physician identified as having life-limiting illness and declining functional state	Practitioner-identified, functional
	Charlson conditions		
Nicholson, 2018	≥2	Healthcare professional thinks patient to be in last year of life, frailty, multiple hospital admissions, increasing uncertainty, deterioration, precarious social support, would benefit from future care planning, requires care coordination, particular conditions	Nature of conditions, healthcare, functional, caregiver burden + practitioner-identified
	Prespecified (not justified)		
Nicholson, 2023	≥2	Aged 60 years or older, practitioner deems them to be in last 1-2 years of life or living with advanced disease or frail	Age + practitioner identified
	Unspecified		
Portz, 2017^b^	≥2	The surprise question, physician thinks life expectancy <1 month and declining functional status	Practitioner-identified, functional
	Charlson conditions		
Powell, 2021	“Multimorbidity”	Older patients, require assistance with activities of daily living	Age, functional
	Unspecified		
Reinke, 2019	“Diagnoses”	Prognostic model – Care assessment needs score	Tool-identified
	Elixhauser conditions		
Schonfeld, 2012^a^	“Not a single terminal diagnosis”	Patient could have term “debility, unspecified” applied	Nature of conditions
	Unspecified		
Stokes, 2017	Charlson >5	Charlson >5	Tool-identified
	Charlson conditions		
The University of Edinburgh, 2022	“Multiple”	Unplanned hospitalisations, poor or deteriorating performance status, dependent on others for care, progressive weight loss, persistent symptoms, the person or family asks for palliative care, best available treatment has poor outcome	Healthcare, functional, caregiver burden, nutritional, symptoms, self-identified
	Unspecified		
The Gold standards framework, 2022	≥2	The surprise question, general physical decline, increasing dependence, repeated unplanned hospitalisations, unstable/deteriorating symptoms, decreased activity, decreasing treatment effectiveness, patient choses to focus on quality of life, >10% weight loss, serious/frequent falls, carer distress, transfer to nursing home, albumin <25g/l, eligible for DS1500 payment, frailty, using multiple services	Practitioner-identified, healthcare, functional, treatment response, nutritional, symptoms, caregiver burden, benefit eligibility, self-identified
	Unspecified		
Wijers, 2019	≥2	Disease burden Morbidity assessment	Tool-identified
	21 chronic conditions from other multimorbidity indexes		

^a^Same description.

^b^Same description.

The International Classification of Diseases (ICD)-9 code “Debility, unspecified”, was used in two different studies.^33,34^ Two studies performed secondary analysis of the same dataset using the same description – serious illness diagnosis with limited life expectancy (1 year or less as per physician assessment), functional impairment at baseline (Australian Karnofksy Performance Status <80%) and multimorbidity (Charlson Comorbidity Index ≥2 conditions)).^35,36^ Of note, this latter description was also used in two further studies.^37,38^ However as this was the same study team and same dataset as another included study^35^ the decision was made to exclude these.

Whilst most studies only used one description, three studies used multiple descriptions (ranging from two to five descriptions) of Advanced Multimorbidity.^27,39,40^

### Types of conditions included in descriptions of advanced multimorbidity

Eighteen of the 44 descriptions used specific conditions in their description of Advanced Multimorbidity. Seven of these restricted their descriptions to only include conditions that were deemed to be ‘life-limiting’ or known to be associated with ‘palliative care need’.^7,17,27,39^ Five of these descriptions used multimorbidity indices – Charlson Comorbidity Index in three,^35,36,41,42^ Elixhauser Comorbidity Index in one^43^ and one description amalgamated a number of indices.^44^ Three descriptions specified that conditions must be non-malignant^31,32,45^ whilst another description specified that participants did not have cancer or end-stage organ failure as any of their conditions.^14^ One description used conditions from the Dartmouth Atlas of Health Care report on Variation of End-of-Life Care.^46^ The final two specified certain conditions, with no explicit justification for how these had been selected.^47,48^

Four descriptions part-specified conditions, e.g. at least one of the conditions had to be of a particular type – two of these, from the same study,^27^ specified that one condition must be life-limiting and two^49,50^ specified participants must have at least one of the most prevalent chronic illnesses in the respective settings.

Additionally, four of the descriptions stipulated that there was no predominant condition which was driving a terminal decline prior to a diagnosis of Advanced Multimorbidity being made.^14,33,34,51,52^

The remaining 22 descriptions did not specify the type of conditions to be included within their description of Advanced Multimorbidity.

**Table:** 

Definitions of multimorbidity by type of conditions
Specific conditions named (n=18/44 papers) Must include ‘life limiting’ or ‘palliative care need’ condition (n=7/44 papers) Includes multimorbidity indices (n=5/44 papers) Specified as non-malignant conditions (n=3/44 papers) Specified as non-malignant and not end-stage organ failure (n=1/44 papers) Dartmouth atlas (n=1/44 papers) Conditions specified but no justification given (n=2/44 papers) Conditions were part specified (n=4/44 papers) Specified there is no predominant condition (n=4/44 papers) No restriction to specific conditions (n=22/44 papers)

### Number of conditions required within descriptions of advanced multimorbidity

Twenty of the total 44 descriptions defined multimorbidity as the presence of two or more conditions.^7,14,27,35,36,39,44,45,47–49,51,53–56^ Four descriptions specified that participants should have three or more conditions.^39,46,50,57^ Two of the studies employed the Charlson index, with one requiring numbers of conditions to be ≥3^41^ and the other requiring >5.^42^ Fifteen descriptions used plural terms, such as ‘multiple’ or ‘several’ conditions, with no associated numerical indicator.^17,31–34,39,43,58–62^ The final three descriptions simply described multimorbidity as ‘multimorbidity’.^58,63,64^

**Table:** 

Definitions of multimorbidity by number of conditions
2 or more conditions (n=20/44 papers) 3 or more conditions (n=4/44 papers) Charlson index + ≥3 conditions (n=1/44 papers) Charlson index + ≥5 conditions (n=1/44 papers) Plural terms used but no specified numerical value (n=15/44 papers) No definition other than ‘multimorbidity’ (n=3/44 papers)

### Approaches to the identification of advanced multimorbidity

Twenty-six descriptions took a multidimensional approach; incorporating a range of different indicators to identify and characterise Advanced Multimorbidity. These comprised between two and nine indicators per description. Palliative care needs assessment tools typically incorporated a greater number of indicators than research studies, with the Supportive and Palliative Care Indicators Tool (SPICT)^62^ using six indicators, *NECesidades PALiativas* (NECPAL)^49,51^ using seven indicators and the Gold Standards Framework Proactive Identification Guidance^56^ using nine indicators. There were 18 descriptions which used only one indicator to identify Advanced Multimorbidity.

### Content analysis of advanced multimorbidity descriptions

Eleven discrete categories of indicators were identified within the 44 included descriptions of Advanced Multimorbidity:

• Global assessments were in 17/44 descriptions, which encompassed holistic assessments of the patient’s health overall
  ○ 12/17 used practitioner assessment
    ■ These included the surprise question (“Would you be surprised if this patient were to die in the next 12 months?”)^17,27,35,36,51,52,56,59,61^; subjective assessments of patients^45,49,51,55^; decisions to undertake future care planning^45,48^ and use of national identification tools^27^
  ○ 4/17 used self-identification
    ■ These involved patients choosing not to pursue curative treatments^51,56,62^ or expressing that death and dying were of an important consideration to them^50^
  ○ 3/17 used assessment tools
    ■ These included the Charlson Comorbidity Index,^42^ Disease Burden Morbidity Assessment^44^ and the Care Assessment Needs tool^43^
• Functional assessments were in 16/44
  ■ These included identifying participants as frail^27,31,32,34,36,39,41,46,48,51,52,56,58,60,62,65^ or measurement overall functional status^39,58^
• Age was in 15/44
  ■ These included studies measuring age alone,^14,33,53,54,60,63,64^ age alongside other indicators^31,32,41,47,48,50,57,59,66–68^ and restricting the study to particular age cut-offs^27,44,51,52^
• Pattern of healthcare utilisation was in 15/44
  ■ These included unscheduled care attendance,^51,56,58,60,62^ hospitalisations^47,51,52,57^ and increased care needs in the community^27,46,51,52,56^
• Type of conditions was in 12/44
  ■ These definitions specified particular conditions which had to be included^7,17,27,33,34,39,45,46,48^
• Nutritional status was in 7/44
  ■ These included measuring weight loss^51,52,56,60,62^ and albumin levels^51,56^
• Symptom burden was in 7/44
  ■ These definitions incorporated symptom assessments^32,46,51,56,60,62,65^
• Caregiver strain was in 5/44
  ■ These definitions measured caregiver strain^48,62^
• Clinical syndromes were in 3/44
  ■ These focussed on particular presentations including pressure ulcers, infections, delirium, dysphagia, falls^51,60^ and acute clinical presentations^45^
• Benefit eligibility was in 2/44
  ■ These focussed on health and welfare benefit eligibility^56,61^
• Responsiveness to treatments was in 2/44
  ■ These incorporated reduced treatment efficacy^56,58^

**Figure 2.**
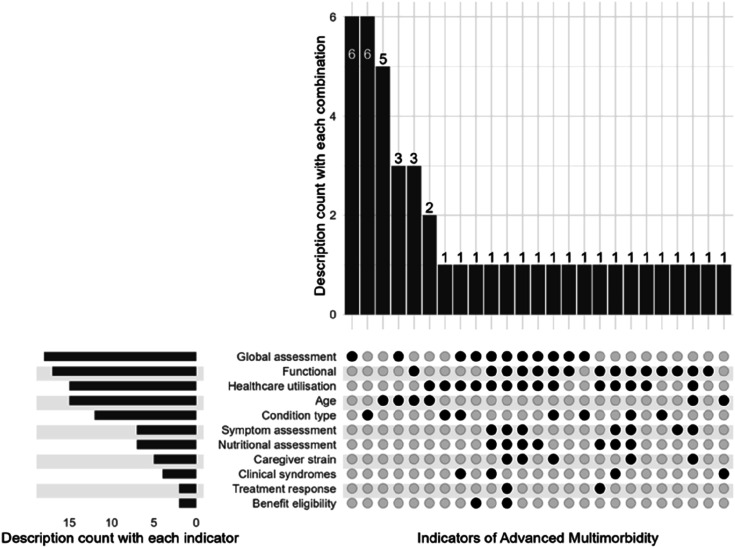
This figure details the various indicators included in the descriptions, with the number of definitions which involved each indicator and the frequency of particular combinations of indicators within the definitions.

Figure 2 summarises the interaction between the various indicators in the descriptions of Advanced Multimorbidity. As this figure shows, the most prevalent use of indicators overall within descriptions was using a single indicator approach of only condition type (n = 6), global assessment (n = 6) and age (n = 6). The most prevalent multidimensional descriptions all featured age plus either functional assessment (n = 3), holistic assessment (n = 3) or pattern of healthcare utilisation (n = 2). The remaining definitions each used different indicators either on their own or in combination.

Supplemental File 2 describes in more detail how each of these indicators were used in the various descriptions of Advanced Multimorbidity.

### Stakeholder consultation

Six public members and thirty-three clinicians and academics participated in stakeholder consultations. Most clinicians and academics felt that there was a role in defining Advanced Multimorbidity – 26 voted ‘yes’, three voted ‘maybe’, and six did not answer. Public advisors all agreed that a description could be useful, but expressed that one description may not suit everyone and were concerned a single description may be too restrictive and may exclude some individuals. Stakeholders felt that a description could be beneficial to a wide range of groups, citing patients, families, bereaved caregivers, healthcare professionals, researchers, healthcare systems and policymakers. Clinicians and academics felt an optimal description should be concise, uncomplicated, contain measurable indicators and ensure some focus on quality of life. Whilst public advisors felt that clinicians should be the ones to drive decisions around identifying Advanced Multimorbidity, they felt it was important that such descriptions were “user-friendly” and accessible and acceptable to patients and those close to them.

## Discussion

The term “Advanced Multimorbidity” was chosen as the basis of this scoping review, recognising that it mirrors widely accepted clinical language used in other end-stage conditions such as advanced cancer^69^ and advanced organ failure^70,71^ and reflects the chronic, incurable nature of such conditions. It has also already been used in previous research.^17,27^ Advances in treatments for both malignant and non-malignant conditions mean that many people are living longer with their incurable conditions,^71,72^ including multimorbidity. Whilst longer lives are often desirable, living with Advanced Multimorbidity is known to carry burdens for patients and their unpaid caregivers.^17^ A palliative care approach has been shown to offer a range of meaningful benefits for people with advanced illness, but the challenge stands about how and when people with multimorbidity, whose burdens may be particularly high, and/or who may be nearing the end of life, can be reliably identified. A palliative care approach is known to benefit many individuals, not only those who are close to the end of their lives – with improvements in quality of life, healthcare utilisation and associated financial burden.^15,73^ However, much of the evidence base for the design and benefits of such palliative care approaches is limited to patients with single or discrete conditions, particularly cancer.^73,74^ The evidence base for the benefits of palliative care approaches in non-malignant and/or multiple conditions is more mixed, with less evident impacts on quality of life.^75^ However, this difference may be more reflective of an underdevelopment of palliative care interventions for people with non-malignant disease and resultant less available evidence for symptom management in such groups, compared to e.g. pain management in cancer.^76^ There remains a further paucity of evidence for implementing palliative care approaches for people with multimorbidity – despite evidence that this group has palliative care needs.^54^ However, prior to developing palliative care interventions that could benefit those with Advanced Multimorbidity, we need to first establish how best to identify individuals who could benefit from such an approach. Understanding current definitions is foundational to this aspiration.

Terminology around multimorbidity is evolving at pace and can be confusing. There is undoubtedly some overlap, in practice, in what is understood to be ‘Advanced Multimorbidity’ and ‘complex multimorbidity’ – with this latter term associated with various working descriptions and ultimately describing when multiple long-term health conditions severely impact health.^77^ It is said that complex multimorbidity can apply at any point in the life course, and indeed it has been used to encompass a wide range of outcomes from work productivity, disability and quality of life. Whilst it is not a term that is used in any consistent way clinically, it has been proposed as a means to identify those who would benefit from multimorbidity-focussed care.^77^ Advanced Multimorbidity, as conceptualised in this review, differs in that it relates to individuals and populations who, by nature of their illness burden and/or proximity to death, may benefit from a palliative care approach.

The publications included in this scoping review were all relatively recent, reflecting both a surge in multimorbidity research and increased awareness of the relevance of palliative care beyond single illnesses such as cancer.^78,79^ There were no end-of-life care guidelines which specifically mentioned how to identify or treat people with multiple long-term conditions, although two palliative care needs assessment tools were identified that specified multimorbidity^56,62^ and one further tool was applied to a population with multimorbidity.^51^ There were few studies which integrated patient and public involvement in their research. This was surprising given the increasing calls from the public, researchers and funding bodies alike to support such activities in recent years, driven by an understanding that collaborative approaches strengthen research relevance, quality and impact.^80^

Age is heavily featured in many of the descriptions of Advanced Multimorbidity in the included studies, perhaps understandably, given that people typically accumulate conditions with advancing age. However, any reliance on age within a working definition could unhelpfully exclude individuals living with Advanced Multimorbidity at a younger age; a phenomenon associated with socioeconomically deprivation, with more deprived individuals shown to both develop multiple long-term conditions earlier^81,82^ and to have poorer health trajectories with earlier onset and more rapid progression of multimorbidity.^83^

There were also descriptions which focussed on particular condition types to describe Advanced Multimorbidity, mainly encompassing conditions typically recognised as associated with palliative care needs, such as cancer and organ failure.^84^ Whilst this disease-focused epidemiological approach has often been adopted in palliative care research, particularly around population estimates,^84^ the feasibility and validity of its prospective application to individuals is unknown. Such a disease-specific approach risks excluding those without ‘typical’ palliative conditions yet with a cumulative disease burden, which means they could benefit from a palliative care approach.

Many of the descriptions included took a multidimensional approach, encompassing a broader, more holistic assessment of patients. The identified palliative care tools (NECPAL,^51^ SPICT^62^ and the Gold Standards Framework^56^) used the highest number of indicators in their descriptions – perhaps reflective of these being utilised in clinical practice rather than in epidemiological research. Many of the commonly used indicators, such as functional assessment, healthcare use and type of condition, can typically be obtained from routinely collated healthcare data, which may have influenced the researchers’ choice in selecting them. There was little emphasis on utilising patient-reported outcomes, such as patient self-identification of advanced illness, symptom assessments and measures of caregiver strain, as a marker of Advanced Multimorbidity. Such assessments have become standard in clinical practice, yet are not routinely utilised for research purposes. In certain fields, such as Oncology, it has been shown that patient-reported outcome measures can be used to identify those who have specialist palliative care needs.^85^ However, proactive use of measures of patient need requires identification that a person may benefit from such an assessment, knowledge of the assessment itself and time and resources to complete the assessment effectively. Nonetheless, our public and clinical stakeholders recommended that future measures of Advanced Multimorbidity should encompass patient and/or caregiver measures relating to quality of life.

The heterogeneity of descriptions described in this review echoes the lack of consistency in how people with multimorbidity are identified in clinical practice.^86^ Greater consensus and standardisation of descriptions of Advanced Multimorbidity would allow for several potential benefits including:

• Identification of people who may benefit from symptom assessments, consideration of their psychological and social needs and future care planning discussions
• Shared decision-making opportunities supporting rationalisation and optimisation of current treatments with resultant reduction in medicalisation (a so-called Realistic Medicine’ approach^87^) for many

Furthermore, this could in turn improve system capacity to deliver preventative and potentially curative treatments for those who would benefit. Such a standardised approach to defining and identifying people with Advanced Multimorbidity would be relevant across a vast range of clinical specialities and teams, including, but not limited to, primary care, specialist palliative care and geriatric medicine, to align their practices in a way that is not currently the case. Future research, including prospective clinical studies and retrospective, epidemiological studies would also benefit from consistency of definition.

## Strengths and limitations

This review was conducted in accordance with scoping review guidance and a pre-published protocol, ensuring it was both comprehensive and robust.^22^ The only deviance from the protocol was in refining the research question to reflect the heterogeneity of studies included and to move away from describing definitions from research, policy and practice, as it was recognised no articles were identifiable from the latter two sources. It captured a wide variety of evidence sources, including Grey Literature. Despite this, no guidelines were identified which focussed specifically on care for people with Advanced Multimorbidity. This may have been due to the study eligibility criteria excluding more general end-of-life care guidelines, as these lacked a specific mention of multimorbidity. Such guidelines may indeed be applicable to those with Advanced Multimorbidity, but were out-with the scope of this review. Furthermore, studies examining multimorbidity (or comorbidity) in specific disease groups were excluded to maximise the generalizability of the findings to those with Advanced Multimorbidity. There may be further lessons to learn about how multimorbidity affects the end-of-life period in people with specific index conditions, such as dementia, cancer or organ failure. Attempts were made to synthesise the findings to provide a usable descriptor for Advanced Multimorbidity. However, the lack of consistency across the included studies was prohibitive. Therefore, this review has focussed on detailing and categorising the various descriptors instead. A further strength of this review is in the incorporation of stakeholder consultations and involvement of our Public Advisory Group from conceptualisation through to output to ensure this work is meaningful and relevant to patients and caregivers.

## Conclusion

This review has highlighted a lack of consistency around how people with Advanced Multimorbidity are defined, described, and thus understood, both in clinical practice and research. Such heterogeneity of approach risks the under-identification of people who may benefit from discussions around future care planning and the consideration of a palliative care approach. In turn, this risks variation in care including poor care experiences and outcomes for many.

The reported definitions which were multidimensional in approach, combining multiple concurrent measures, offer considerable appeal and may be more likely to lead to a greater number of people being identified, whilst also supporting a more holistic assessment of individuals’ needs.

Further research in this important area is a necessity if we are to ensure that people living with Advanced Multimorbidity are to access the palliative care they need. Such research must include input from a variety of stakeholders, including people with lived experience, clinicians, researchers and policymakers. Future work could look to evaluate the predictive value of existing descriptions and develop novel multidimensional descriptors with person-centred, measurable variables included, building on the evidence gathered by this review. This could be aided further by consideration of multimorbidity as a broad concept, instead of distilling this down to discrete conditions co-existing. Furthermore, validation of future ways to identify those with Advanced Multimorbidity should be undertaken in diverse populations. Once robust methods of identifying Advanced Multimorbidity are established, the challenge for researchers, policymakers and clinicians lies in developing robust palliative care interventions fit to meet the needs of this diverse group. Ultimately, future work needs to be focused on delivering improvements in care as, in the words of a member of the studies’ Public Advisory Group: “it’s not just about defining something, it’s about improving the end of life for people”.

## Supplemental Material

Supplemental Material - Descriptions of advanced multimorbidity: A scoping review with content analysis Supplemental Material for Descriptions of advanced multimorbidity: A scoping review with content analysis by Sarah P Bowers, Polly Black, Lewis McCheyne, Darcy Wilson, Rose S Penfold, Liam Stapleton, Pam Channer, Sarah E E Mills, Linda Williams, Frances Quirk, and Jo Bowden in Journal of Multimorbidity and Comorbidity
