# Supplementary material for: Descriptions of advanced multimorbidity: A scoping review with content analysis
Source: J Multimorb Comorb. 2025 Mar 18;15:26335565251326309. doi: 10.1177/26335565251326309 (PMC11920996; doi:10.1177/26335565251326309)
Supplement: Supplemental Material - Descriptions of advanced multimorbidity: A scoping review with content analysis [file sj-pdf-1-cob-10.1177_26335565251326309.pdf]

Supplementary File 1

Search strategies used for each database

Medline and Embase

1. Multimorbidity/
2. (multimorbid* or multi-morbid* or polymorbid* or poly-morbid* or multicondition* or multi-condition* or ((multiple or coexist* or co-exist* or concurrent or co-occur*) adj (comorbid* or co-morbid* or "long term" or long-term or chronic or disease* or illness* or diagnos* or morbid* or condition*))).ti,ab
3. 1 or 2
4. Palliative Care/
5. Terminal Care/
6. (palliat* or terminal or "end of life" or end-of-life or dying or died or "last year" or "final year" or ((final or last) adj1 month*)).ti,ab.
7. 4 or 5 or 6
8. 3 and 7
9. (("end stage" or end-stage or "advanc*" or "complex") adj2 (multimorbid* or multi-morbid* or polymorbid* or poly-morbid* or multicondition* or multi-condition* or ((multiple or coexist* or co-exist* or concurrent or co-occur*) adj (comorbid* or co-morbid* or "long term" or long-term or chronic or disease* or illness* or diagnos* or morbid* or condition*)))).ti,ab
10. 8 or 9

CINAHL

1. (MH "Comorbidity")
2. TI ((multimorbid* or multi-morbid* or polymorbid* or poly-morbid* or multicondition* or multi-condition* or ((multiple or coexist* or co-exist* or concurrent or co-occur*) W1 (comorbid* or co-morbid* or "long term" or long-term or chronic or disease* or illness* or diagnos* or morbid* or condition*)))) OR AB ((multimorbid* or multi-morbid* or polymorbid* or poly-morbid* or multicondition* or multi-condition* or ((multiple or coexist* or co-exist* or concurrent or co-occur*) W1 (comorbid* or co-morbid* or "long term" or long-term or chronic or disease* or illness* or diagnos* or morbid* or condition*))))
3. S1 OR S2
4. (MH "Palliative Care")
5. (MH "Terminal Care")
6. TI ((palliat* or terminal or "end of life" or end-of-life or dying or died or "last year" or "final year" or ((final or last) W1 month*))) OR AB ((palliat* or terminal or "end of life" or end-of-life or dying or died or "last year" or "final year" or ((final or last) W1 month*)))
7. S4 OR S5 OR S6
8. S3 AND S7
9. TI ((("end stage" or end-stage or "advanc*" or "complex") W2 (multimorbid* or multi-morbid* or polymorbid* or poly-morbid* or multicondition* or multi-condition* or ((multiple or coexist* or co-exist* or concurrent or co-occur*) W1 (comorbid* or co-morbid* or "long term" or long-term or chronic or disease* or illness* or diagnos* or morbid* or condition*))))) OR AB ((("end stage" or end-stage or "advanc*" or "complex") W2

(multimorbid* or multi-morbid* or polymorbid* or poly-morbid* or multicondition* or multi-condition* or ((multiple or coexist* or co-exist* or concurrent or co-occur*) W1 (comorbid* or co-morbid* or "long term" or long-term or chronic or disease* or illness* or diagnos* or morbid* or condition*)))))

10. S8 or S9

Scopus

(TITLE-ABS-KEY ((("end stage" OR end-stage OR "advanc*" OR "complex") PRE/2 (multimorbid* OR multi-morbid* OR polymorbid* OR poly-morbid* OR multicondition* OR multi-condition* OR ((multiple OR coexist* OR co-exist* OR concurrent OR co-occur*) PRE/1 (comorbid* OR co-morbid* OR "long term" OR long-term OR chronic OR disease* OR illness* OR diagnos* OR morbid* OR condition*)))))) OR ((TITLE -ABS-KEY ((multimorbid* OR multi-morbid* OR polymorbid* OR poly-morbid* OR multicondition* OR multi-condition* OR ((multiple OR coexist* OR co-exist* OR concurrent OR co-occur*) PRE/1 (comorbid* OR co-morbid* OR "long term" OR long-term OR chronic OR disease* OR illness* OR diagnos* OR morbid* OR condition*))) .)) AND (TITLE -ABS-KEY ((palliat* OR terminal OR "end of life" OR end-of-life OR dying OR died OR "last year" OR "final year" OR ((final OR last) PRE/1 month*))))))

APA PsychInfo

1. Exp Comorbidity
2. (multimorbid* or multi-morbid* or polymorbid* or poly-morbid* or multicondition* or multi-condition* or ((multiple or coexist* or co-exist* or concurrent or co-occur*) adj (comorbid* or co-morbid* or "long term" or long-term or chronic or disease* or illness* or diagnos* or morbid* or condition*))).ti,ab
3. 1 or 2
4. Exp Palliative Care/
5. Exp Terminally Ill Patients/
6. (palliat* or terminal or "end of life" or end-of-life or dying or died or "last year" or "final year" or ((final or last) adj1 month*)).ti,ab.
7. 4 or 5 or 6
8. 3 and 7
9. (("end stage" or end-stage or "advanc*" or "complex") adj2 (multimorbid* or multi-morbid* or polymorbid* or poly-morbid* or multicondition* or multi-condition* or ((multiple or coexist* or co-exist* or concurrent or co-occur*) adj (comorbid* or co-morbid* or "long term" or long-term or chronic or disease* or illness* or diagnos* or morbid* or condition*)))).ti,ab
10. 8 or 9

Supplementary File 2

Details of different indicators identified through content analysis.

Content Analysis Process

Content analysis supports a flexible approach, and for the purposes of this review there were three main phases:

- Preparation phase: the description was selected from the data sources, identifying key words and phrases used, alongside a description of the context
- Organisation phase: open coding was used to create broad headings describing the descriptions
- Abstraction phase: an extrapolated description of the descriptions was formulated through further categorisation, enabling a narrative description[29, 30].

Global assessments

There were 17 descriptions which involved global, holistic assessments of individuals to describe Advanced Multimorbidity, with some descriptions doing this in multiple ways.

Twelve of these 17 descriptions used practitioner assessment to identify Advanced Multimorbidity[17, 27, 35, 36, 45, 48, 49, 51, 52, 55, 56, 59, 61]. Of these twelve, eight descriptions used the surprise question ("Would you be surprised if this patient were to die in the next 12 months?") as an identifier of Advanced Multimorbidity[17, 27, 35, 36, 51, 52, 56, 59, 61]. Three descriptions asked for clinicians' subjective assessments that participants had an advanced illness[45, 49, 51, 55]. Two descriptions incorporated future care plans (e.g. do not attempt resuscitation (DNR) forms) to identify Advanced Multimorbidity[45, 48]. One description additionally highlighted the Gold Standards Framework criteria, a UK national guidance to aid clinicians to identify patients who may be approaching the end of life[27].

Four of these descriptions included patient self-identification[50, 51, 56, 62]. Three of these, all guidelines, incorporated individuals choosing not to pursue curative treatments and/or to focus on quality of life[51, 56, 62]. One qualitative study, employing secondary analysis, identified older adults with multiple chronic conditions who had raised issues around death and dying in a previous qualitative study, thus highlighting this was an important focus for them[50].

Three descriptions used assessment tools with numerical cut-offs to identify Advanced Multimorbidity[42-44]. One study used the Charlson with a score of >5 representing end-of-life for people with multimorbidity[42]. Another used the Disease Burden Morbidity Assessment which has been shown to correlate with survival, with scores of ≥ 10 representing higher risk of death alongside higher disease burden[44]. The Care Assessment Needs tool was used by another study to identify those with multimorbidity who had a high level of need[43].

Functional assessments

Functional assessments were incorporated within 16/44 descriptions[27, 31, 32, 34, 36, 39, 41, 46, 48, 51, 52, 56, 58, 60, 62, 65]. Many used frailty scores to identify functional decline including Barthel[51, 56], ECOG[51], Karnofsky Prognostic Scores[27, 35, 36, 51], Clinical Frailty Scale[32], and Electronic Frailty Index[48]. Others opted for descriptors of a declining functional performance status with regards to changes in management of activities of daily living[46, 52, 56, 60, 62, 65, 66]. Others simply described functional declines as "frailty"[27, 31, 41, 56]. One of the descriptions operationalised functional assessment via read-codes "13CA Housebound AND multimorbidity"[39]. One description caveated a change in functional status alongside symptoms related to this: "declining functional status accompanied by increasing fatigue"[58].

Age

Fifteen descriptions incorporated age within their description of Advanced Multimorbidity. Five of these used age alone to identify a population with multimorbidity should be considered as potentially approaching the end of life [14, 33, 53, 54, 60, 63, 64]. Nine descriptions used age as part of their description alongside one other marker –functional status in three[31, 41, 66], predicted deterioration in three[50, 55, 59], healthcare use in two[47, 57] and discharge from intensive care unit in one[67]. One description incorporated age alongside multiple other indicators[32]. The ages used in these descriptions ranged from ≥50 years[47] to ≥80 years[63]. Four other studies did not use age as part of their description but did restrict their study population to those within certain age cut-offs[27, 44, 51, 52].

Healthcare utilisation

Healthcare utilisation was incorporated in 15/44 descriptions[27, 32, 45-48, 51, 52, 56-58, 60-62]. Many had a focus on unscheduled care services, particularly attendance at accident and emergency and unplanned hospital admissions [51, 56, 58, 60, 62]. Some studies gave a numerical cut-off to identify higher healthcare use – two or more hospital admissions in the last year was used in three descriptions [47, 51, 52] and three or more in one description[57]. The timings of these hospitalisations varied from 6 months [52] and 12 months[47, 51, 57]. There were only five descriptions which considered increased care needs in the community[27, 46, 51, 52, 56].

Type of Conditions

There were 12 descriptions which incorporated particular types of conditions to be reflective of Advanced Multimorbidity[7, 17, 27, 33, 34, 39, 45, 46, 48]. Seven of these descriptions included conditions that were life-limiting or associated with perceived palliative care needs including cancer, organ failure, dementia and other progressive neurological conditions[7, 17, 27, 39]. Two studies selected conditions based on population data around the most common causes of death[45, 46]. Another description included a list of conditions from which participants should have two or more,

however did not justify further the choice of these[48]. The ICD-10 code “Debility, unspecified” was applied in two studies to identify groups of patients with multiple illnesses but who did not have a dominant condition that could account for their terminal decline.[33, 34].

Declining nutritional status

Nutritional assessment was incorporated into seven descriptions[46, 51, 52, 56, 58, 60, 62]. Four descriptions quantified this by specifying that weight loss should be >10%[51, 52, 56, 60], one further study did not quantify the amount of weight loss but stated this should be progressive[62]. In two descriptions, nutritional assessments were captured through biochemical means as low serum albumin[51, 56].

Symptom Assessment

Seven descriptions utilised symptom assessment to help to identify individuals with Advanced Multimorbidity[32, 46, 51, 56, 60, 62, 65]. Most of these took the focus of unresolved symptoms, without specifying the particular types of symptoms that should be included[32, 56, 60, 62, 65]. However, one description took a focus on “emotional distress”[51] and another that “pain limits activities”[46].

Caregiver strain

Five studies featured caregiver strain as a consideration in their Advanced Multimorbidity description[32, 46, 48, 56, 62]. This was conceptualised in different ways with some studies referring to caregiver wellbeing, considering their distress or suffering[32, 56] or by identifying financial, emotional or physical concerns[46]. Others identified caregiver strain as an increasing pressure on caregivers time, as the patient got less well[48, 62].

Presence of specific syndromes

There were three descriptions which identified particular clinical conditions or presentations as indicating Advanced Multimorbidity[45, 51, 60]. One study identified that having two or more of “persistent pressure ulcers (stage III-IV); recurrent infections (>1); delirium; persistent dysphagia; falls (>2)” in the last 6 months, was indicative of illness severity[51]. Acute events in the context of multiple chronic conditions was deemed to be indicative of advanced illness in another study[45]. Both non-healing wounds and recurrent infections were deemed to be of importance in one of the included case studies[60].

Benefit eligibility

Health and welfare benefit eligibility was part of description in two separate studies, with the type of benefit reflecting the underlying health and welfare system of the country of origin of those studies[56, 61]. One study exploring different healthcare providers within the United States of America included “Medicare Advantage and Medicare Shared Savings patients” as part of their description[61]. The Gold Standards Framework Proactive Identification Guidance, guidelines designed for implementation in the United Kingdom incorporated “Considered eligible for DS1500 payment” – a benefit that is only available to patients who are deemed to be terminally ill[56].

Treatment Response

Two descriptions incorporated reduced treatment efficacy to identify individuals with Advanced Multimorbidity[56, 58]. These both considered a reduction in response to treatments as a marker of a point where there was less likelihood of reversibility.
